# Prevalence and genotype distribution of HPV infection among 214,715 women from Southern China, 2012–2018: baseline measures prior to mass HPV vaccination

**DOI:** 10.1186/s12879-021-06019-5

**Published:** 2021-04-07

**Authors:** Li-pei Luo, Ping He, Qiao-tu Liu, Yang-hua Jiang, Yang-nan Zhang, Qing-zhao Li, Qiu Li, Sheng-tao Li, Fan Yang, Hua Ling, Xin-gui Dai, Zhong-yu Li, Hong-liang Chen

**Affiliations:** 1 The First School of Clinical Medicine, Southern Medical University, Chenzhou NO.1 People’s Hospital, Chenzhou, 423000 P.R. China; 2 Institute of Pathogenic Biology, Hengyang Medical College, Hunan Provincial Key Laboratory for Special Pathogens Prevention and Control, Hunan Province Cooperative Innovation Center for Molecular Target New Drug Study, University of South China, Hengyang, 421001 P.R. China

**Keywords:** Human papillomavirus, High-risk HPV, Low-risk HPV, Prevalence, Genotype, Single HPV infection, Multiple HPV infection

## Abstract

**Background:**

The epidemiology on the human papillomavirus (HPV) among females in Southern China is not well-established. Baseline data on the prevalence of HPV infection in China prior to mass prophylactic HPV vaccination would be useful. Thus, this study aims to determine the type-specific HPV prevalence and distribution among females from Southern China prior to mass HPV vaccination.

**Methods:**

A retrospective cross-sectional study employing 214,715 women attending ChenZhou NO.1 People’s Hospital for cervical screening during 2012–2018 was conducted prior to widespread HPV vaccination. HPV genotype was detected using nucleic acid molecular diversion hybridization tests. The overall prevalence, age-specific prevalence, type distribution, and annual trend were analyzed.

**Results:**

The overall HPV prevalence was 18.71% (95% confidence interval [CI], 18.55–18.88%) among Southern China females. During 2012–2018, the prevalence of HPV infection showed a downward tendency, from 21.63% (95% CI, 21.07–22.20%) in 2012 to 18.75% (95% CI, 18.35–19.16%) in 2018. Age-specific HPV distribution displayed a peak at young women aged less than 21 years (33.11, 95% CI, 31.13–35.15%), 20.07% (95% CI, 19.70–20.44%) among women aged 21–30 years, 17.29% (95% CI, 17.01–17.57%) among women aged 31–40 years, 17.23% (95% CI, 16.95–17.51%) among women aged 41–50 years, 21.65% (95% CI, 21.11–22.20%) among women aged 51–60 years, and 25.95% (95% CI, 24.86–27.07%) among women aged over 60 years. Of the 21 subtypes identified, the top three prevalent high-risk HPV (HR-HPV) genotypes were HPV52 (5.12%; 95% CI, 21.11–22.20%), − 16 (2.96%; 95% CI, 2.89–3.03%), and − 58 (2.51%; 95% CI, 2.44–2.58%); the predominant low-risk HPV (LR-HPV) genotypes were HPV81 (1.86%; 95%CI, 1.80–1.92%) and − 6 (0.69%; 95% CI, 0.66–0.73%) respectively. Incidence of HR-HPV only, LR-HPV only and mixed LR- and HR-HPV were 15.17, 2.07 and 1.47% respectively. Besides, single HPV infection accounted for 77.30% of all positive cases in this study.

**Conclusions:**

This study highlights 1) a high prevalence of HPV infection among females with a decreasing tendency towards 2012–2018, especially for young women under the age of 21 prior to mass HPV vaccination; 2) HPV52, − 16 and − 58 were the predominant HPV genotypes, suggesting potential use of HPV vaccine covering these HPV genotypes in Southern China.

## Background

Human papillomavirus (HPV) is the leading cause of cervical cancer and responsible for about 91% of anal cancers, 75% of vaginal cancers and 70% oropharynx cancer [1]. It is estimated that there were over 528,000 new cervical cancer patients and 266,000 death worldwide recorded in 2012, among which 85% occurred in developing countries [2]. In China, 130,000 new cases of cervical cancer are diagnosed annually, especially in young women within the first few years after sexual debuts [3, 4]. The morbidity and mortality of cervical cancer in developed countries has fallen over the last two decades because of an enhanced awareness of cervical cancer prevention and effective screening [5]. However, infection rate of HPV has fluctuated and not shown a downward trend, indicating a relatively heavy burden of HPV infection in China [6, 7].

HPV genotypes are linked to the degree of cervical lesions [6]. Infections with low-risk HPV (LR-HPV) types, such as HPV6 and − 11, cause benign or low-grade changes in cervix cells, genital warts, and recurrent respiratory papillomatosis. Contrarily, high-risk HPV (HR-HPV) type cause cervical, anal, and other genital cancers, which could be detected in 99% of cervical cancers—the second most common cancer in women worldwide. HPV16 and − 18 have been found to be the most pathogenic HR-HPV types, causing about 70% of cervical cancers worldwide [7–10]. Of note, most HPV infections are completely asymptomatic, resulting in a delay in diagnosis and follow-up treatment with disastrous consequences [11]. Moreover, HPV-infected individuals without early intervention will develop long-lasting HPV infections that put them at risk for cervical cancer, and increase the risk of sexual transmission to their partners, resulting in approximately estimated $1.7 billion in direct medical costs annually in the United States [12].

The prevalence of HPV varies geographically widely, ranging from 6% in southeastern Asia to 32% in eastern Africa, and from 6.7 to 44.5% in China [13]. HPV screening and vaccine are considered being the most effective measures for preventing HPV infection. HPV vaccine has been clinically applied for more than 10 years [14], however, officially launched on July 2017 in mainland China and available mainly in the developed regions in China currently, such as Beijing, Shanghai, and Guangzhou. Besides, the HPV vaccine made in China was approved in 2020. Representative data on type-specific prevalence of HPV infection in China could provide a baseline estimation to the burden of HPV infection and could help guiding programs on HPV-based cervical cancer screening and strategies on vaccine-based HPV prevention. To date, the existing data about epidemiological characteristics of HPV infection in Southern China are still scantly, especially lacking large sample study on the genotype prevalence of HPV. Therefore, this paper was conducted to determine the prevalence and type distribution of HPV among 214,715 females in Southern China, including its trends from 2012 to 2018 and age-specific prevalence. Such data provide a baseline pre-vaccine population-based prevalence of HPV in Southern China, and help guiding models evaluating impact and cost-effectiveness by comparison between future vaccinated populations, and implementation of the prophylactic HPV vaccine according to the specific types’ distribution in the region.

## Methods

### Study population

From January 2012 to December 2018, 214,715 females (age range from 19 to 83 years) were enrolled in this study. All participants were from gynecology department and physical examination center of Chenzhou NO.1 People’s Hospital. They have got HPV tests for various reasons, including physical examination, vaginitis, urethritis, irregular vaginal bleeding, cervicitis, undiagnosed abdominal pain and genital warts. In addition, repeated samples from the same women were excluded.

### Cervical specimen collection and management

All of the cervical samples were taken by the physician, but not self-sampling, using a 200 mm polyethylene Cervix brush device (Hybribio Corp, Guangdong) following the regular procedures for speculum examination. The samples were transferred to a sampling tube containing a transport medium (Hybribio Corp, Guangdong) and stored at 2–8 °C until HPV DNA extraction within a week. Cervical samples can be kept in a transport medium for 2 weeks at 4 °C according to the manufacturer’s manual.

### HPV genotyping

DNA was extracted by Cell Lysis Kit (Hybribio Corp, Guangdong) accompanying with the negative and positive quality control products throughout the whole process. HPV genotyping have been performed using HPV assay kit (Hybribio Corp, Guangdong) since 2012 with no modifications. This kit detected 21 HPV genotype via gene amplification technology and diversion hybridization. The low limit of detection (LOD) for HPV DNA is 500 copies/ml according to manufacturer’s protocol. This assay uses HPV L1 consensus PCR primers (MY09/11) for the amplification and a human housekeeping gene β-globin as an endogenous internal control to ensure appropriate DNA purification, PCR reaction and specimen quality. PCR was carried out in 25 μl reaction mixture in a thermal cycler, the cycling parameters of which were as follows: 20 °C for 10 min, 95 °C for 9 min, followed by 40 cycles (20s at 95 °C, 30 s at 55 °C and 30 s at 72 °C), with a final extension at 72 °C for 5 min. HPV types were then classified via a nylon membrane immobilized with 21 different type-specific probes. Quality controls of HPV genotyping including External Quality Assessment and Internal Quality Control were implemented throughout the study.

### Statistical analysis

Analyses were conducted with SPSS version 19.0. Descriptive statistical analysis was performed on the distribution of HPV genotypes using indicators such as frequency and prevalence. The Chi-square test was used to test the differences between prevalence, genotypes and number of co-infections of HPV in different age groups and time groups.

### Ethical consideration

The study was approved by the ethics Committee of Chenzhou NO.1 People’s Hospital and conducted strictly in accordance with the Declaration of Helsinki, including the confidentiality and anonymity. No informed consent or other action on the part of the patients was required due to anonymous analyses of the data. All experiments were carried out in the lab certified by the National Center for Clinical Laboratories following the laboratory biosafety guidelines.

## Results

### Overall prevalence and genotype distribution of HPV

**Fig. 1:**
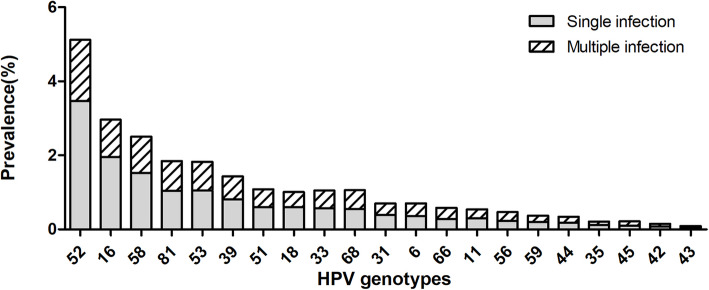
Prevalence of HPV infections. **a**: Prevalence of HPV infections among females, 2012–2018; **b**: genotype-specific distribution of HPV infections, 2012–2018. The six top prevalent HPV genotypes were HPV52 (5.12%), −16 (2.96%), −58 (2.51%), −53 (1.84%), −39 (1.43%), and − 51 (1.08%) respectively. HPV genotypes, except for HPV51, − 39 and − 53, presented a tendency of decrease from 2012 to 2018

From 2010 to 2018, 214,715 eligible female participants were enrolled for final statistical analysis in this study. Among them, 40,168 cases (18.71%; 95% CI, 18.55–18.88%) were positive for at least one HPV genotype (Table 1). A general decline in prevalence was observed over the years from 21.63% (95%CI, 21.07–22.20%) in 2012 to 18.75% (95% CI, 18.35–19.16%) in 2018. The prevalence of single and multiple HPV infections, as well as HPV genotypes except for HPV51, − 39 and − 53 also showed a similar decreasing trend (Fig. 1a, b).

**Table 1:** Overall prevalence of HPV genotype in single and multiple infections

Genotypes	Single infection		Multiple infections		Total	
	(n, %)	95% CI	(n, %)	95% CI	(n, %)	95% CI
HR-HPV*						
52	7457(3.47)	3.39–3.55	3537(1.65)	1.60–1.70	10,994 (5.12)	5.03–5.21
16	4183 (1.95)	1.89–2.01	2175 (1.01)	0.97–1.05	6358 (2.96)	2.89–3.03
58	3267 (1.52)	1.47–1.57	2128 (0.99)	0.95–1.03	5395 (2.51)	2.44–2.58
53	2261 (1.05)	1.01–1.09	1683 (0.78)	0.74–0.82	3944 (1.84)	1.78–1.90
39	1747 (0.81)	0.77–0.85	1330 (0.62)	0.59–0.65	3077 (1.43)	1.38–1.48
51	1284 (0.60)	0.57–0.63	1030 (0.48)	0.45–0.51	2314 (1.08)	1.04–1.12
68	1187 (0.55)	0.52–0.58	1092 (0.51)	0.48–0.54	2279 (1.06)	1.02–1.10
33	1231 (0.57)	0.54–0.60	1031 (0.48)	0.45–0.51	2262 (1.05)	1.01–1.09
18	1279 (0.60)	0.57–0.63	878 (0.41)	0.38–0.44	2157 (1.00)	0.96–1.04
31	841 (0.39)	0.36–0.42	664 (0.31)	0.29–0.33	1505 (0.70)	0.67–0.74
66	598 (0.28)	0.26–0.30	639 (0.30)	0.28–0.32	1237 (0.58)	0.55–0.61
56	501 (0.23)	0.21–0.25	520 (0.24)	0.22–0.26	1021 (0.48)	0.45–0.51
59	436 (0.20)	0.18–0.22	368 (0.17)	0.15–0.19	804 (0.37)	0.35–0.40
45	225 (0.10)	0.09–0.11	258 (0.10)	0.11–0.14	483 (0.22)	0.20–0.24
35	258 (0.12)	0.11–0.14	200 (0.09)	0.08–0.10	458 (0.21)	0.19–0.23
**LR- HPV**^**#**^						
81	2240 (1.04)	1.00–1.08	1743 (0.81)	0.77–0.85	3983 (1.86)	1.80–1.92
6	765 (0.36)	0.34–0.39	723 (0.34)	0.32–0.37	1488 (0.69)	0.66–0.73
11	643 (0.30)	0.28–0.32	519 (0.24)	0.22–0.26	1162 (0.54)	0.51–0.57
44	380 (0.18)	0.16–0.20	342 (0.16)	0.14–0.18	722 (0.34)	0.32–0.37
42	173 (0.08)	0.07–0.09	148 (0.07)	0.06–0.08	321 (0.15)	0.13–0.17
43	92 (0.04)	0.03–0.05	105 (0.05)	0.04–0.06	197 (0.09)	0.08–0.10
**Total**	31,048 (14.46)	14.31–14.61	9120 (4.25)	4.17–4.34	40,168 (18.71)	18.55–18.88

Single infection versus multiple infections considering HR-HPV *P < 0.001 and LR- HPV ^**#**^*P* < 0.001. *HPV* Human papillomavirus, *HR-HPV* High-risk HPV, *LR-HPV* Low-risk HPV, *CI* Confidence interval

**Fig. 2:**
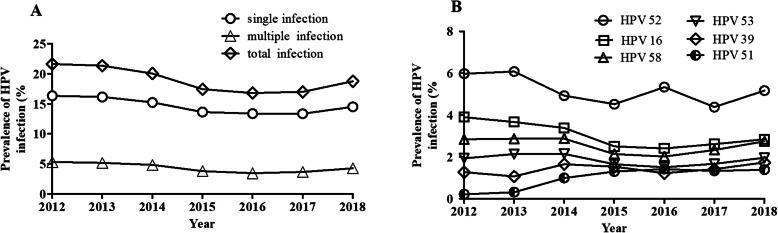
Prevalence of HPV genotypes in single and multiple infections. Fifteen HR-HPV genotypes: HPV52, − 16, − 58, − 53, − 39, −51, −68, −33, −18, −31, − 66, −56, −59, −45 and − 35; six LR-HPV genotypes: HPV81, − 6, − 11, − 44, − 42 and − 43

HPV52 (5.12%; 95% CI, 5.03–5.21%) was the most common genotype, followed by HPV16 (2.96%; 95% CI, 2.89–3.03%) and − 58 (2.51%; 95% CI, 2.44–2.58%), accounting for 76.36% of all detected HPV types (Table 1), which were also the top three prevalent genotypes among HR-HPV, while its counterpart LR-HPV was HPV81, − 6 and − 11, respectively (Table 1 and Fig. 2).

### Prevalence of high-risk, low-risk HPV by age and types

The overall HR-HPV and LR-HPV positive rates were 20.63% (95% Cl 20.46–21.80%) and 3.67% (95% Cl 3.59–3.75%) respectively. HR-HPV infection was significantly higher among women with single infection (12.46%) than those with multiple HPV infections (8.17%, *P* < 0.001), and a similar result was also observed for LR- HPV infection (2.0% vs. 1.67%, *P* < 0.001, Table 1). When dividing the subjects into six age groups (< 21, 21–30, 31–40, 41–50, 51–60 and > 60 years), we found the highest prevalence of HPV infection was observed among females under 21 years old (33.11%; 95% CI, 31.13–35.15%), while the lowest HPV prevalence occurred in women aged 41–50 years (17.23%; 95% CI, 16.95–17.21%). There was a statistically significant difference in HPV prevalence among the participants from different age groups (*P* < 0.001, Table 2). Furthermore, age-specific HPV distribution showed a bimodal pattern. Two peaks of HPV infection were detected in the population of the youngest group (33.11%) and the oldest group (25.95%), suggesting young women may be more prone to HPV infection.

**Table 2:** Age-specific distribution of HPV infection

	Age, years (n, %)							***P***
	< 21 (***n*** = 2111)	21–30 (***n*** = 45,211)	31–40 (***n*** = 70,681)	41–50 (***n*** = 68,488)	51–60 (***n*** = 22,142)	> 60 (***n*** = 6082)	Total	
**HPV positive numbers**	699 (33.11)	9073 (20.07)	12,223 (17.29)	11,801 (17.23)	4794 (21.65)	1578 (25.95)	40,168 (18.71)	< 0.001
**Number of co-infections**								
single HPV genotype	411 (19.47)	6818 (15.08)	9795 (13.86)	9464 (13.82)	3519 (15.89)	1041 (17.12)	31,048 (14.16)	< 0.001
double HPV genotypes	167 (7.91)	1671 (3.70)	1957 (2.77)	1882 (2.75)	906 (4.09)	343 (5.64)	6926 (3.23)	
triple HPV genotypes	74 (3.51)	438 (0.97)	349 (0.49)	350 (0.51)	257 (1.16)	121 (1.99)	1589 (0.74)	
Quadruple HPV genotypes	32 (1.52)	108 (0.24)	90 (0.13)	78 (0.11)	74 (0.33)	45 (0.74)	427 (0.20)	
≥ five HPV genotypes	15 (0.71)	38 (0.08)	32 (0.05)	27 (0.04)	38 (0.17)	28 (0.46)	178 (0.08)	
**HPV genotype**								
HR-HPV only	423 (20.04)	7122 (15.75)	10,146 (14.35)	9742 (14.22)	3881 (17.53)	1259 (20.70)	32,573 (15.17)	< 0.001
LR-HPV only	114 (5.40)	1123 (2.48)	1309 (1.85)	1293 (1.89)	484 (2.19)	125 (2.06)	4448 (2.07)	
mixed LR- and HR-HPV	162 (7.67)	828 (1.83)	768 (1.09)	766 (1.12)	429 (1.94)	194 (3.19)	3147 (1.47)	

*HPV* Human papillomavirus, *HR-HPV* High-risk HPV, *LR-HPV*, Low-risk HPV, *CI* Confidence interval

The prevalence of HR-HPV only, LR-HPV only, and mixed LR- and HR-HPV infections were 15.17% (95% CI, 15.02–15.32%), 2.07% (95% CI, 2.01–2.13%), 1.47% (95% CI, 1.42–1.52%), respectively, and a significant difference was observed in the distribution of HR-HPV only, LR-HPV only, and mixed LR- and HR-HPV infections among the six age groups (*P* < 0.001, Table 2). The highest prevalence of HR-HPV only infection (20.04%) was still found in the youngest age group, and it decreased gradually as the age increased (21–30, 31–40 and 41–50 years) before hitting another increasing trend in the older age groups (51–60 and > 60 years), showing a bimodal curve. A similar age-specific prevalence pattern was also noted in the LR-HPV only, and mixed LR- and HR-HPV infections.

### Prevalence of single and multiple HPV infections

The prevalence of single HPV infection was 14.46% (14.46%; 95% CI, 14.31–14.61%) accounting for 77.30% of the 40,168 HPV-positive cases, whereas multiple infections (4.25%; 95% CI, 4.17–4.34%) were rare. For those infected with multiple HPV genotypes, the prevalence decreased significantly as the number of infected HPV genotypes increased (*P* < 0.001, Table 2). Notably, four participants were found to be infected with nine HPV types (data not shown).

In addition, there was a significant difference in the distribution of single and multiple HPV infections among different age groups (*P* < 0.001, Table 2). Young women aged less than 21 years had the highest prevalence of both single (19.47%) and multiple HPV infection (13.64%), which further confirmed our previous finding [15] of a high susceptibility to HPV infection in the young population.

### Prevalence of LR-HPV only, HR-HPV only, and mixed LR- and HR-HPV infections by number of HPV genotypes

A significantly different distribution was observed for LR-HPV only, HR-HPV only, and Mixed LR- and HR-HPV infections among women co-infected with a different number of HPV genotypes (*P* < 0.001). Both LR- and HR-HPV only infection had the highest prevalence in single HPV infection (2.00% vs. 12.46%), which declined gradually as the number of co-infections increased. Particularly, HR-HPV only infection took up the majority in women with double and triple HPV infection, and mixed LR- and HR-HPV infections correlated with those infected with more than three HPV genotypes, while none of the infections with three or more genotypes were detected in those with LR-HPV only infections (Table 3). These data indicated that LR-HPV only infections primarily occurred in single HPV infections, while mixed HR-HPV infections in multiple HPV infections.

**Table 3:** Prevalence of LR-HPV only, HR-HPV only, and Mixed LR- and HR-HPV infections in women co-infection with different numbers

Number of co-infections	LR-HPV only		HR-HPV only		mixed LR- and HR-HPV		Total	
	n, (%)	95% CI	n, (%)	95% CI	n, (%)	95% CI	n, (%)	95% CI
Total	4448 (2.07)	2.01–2.13	32,571 (15.17)	15.02–15.32	3149 (1.47)	1.42–1.52	40,168 (18.71)	18.55–18.88
Single HPV genotype	4293 (2.00)	1.94–2.06	26,755 (12.46)	12.32–12.60	NA	NA	31,048 (14.46)	14.31–14.61
Double HPV genotypes	152 (0.07)	0.06–0.08	4752 (2.21)	2.15–2.27	2022 (0.94)	0.90–0.98	6926 (3.23)	3.16–3.31
Triple HPV genotypes	3 (0.00)	NA	849 (0.40)	0.37–0.43	737 (0.34)	0.32–0.37	1589 (0.74)	0.70–0.78
Quadruple HPV genotypes	0 (0.00)	NA	169 (0.08)	0.07–0.09	258 (0.12)	0.11–0.14	427 (0.20)	0.18–0.22
≥Five HPV genotypes	0 (0.00)	NA	46 (0.02)	0.01–0.03	132 (0.06)	0.05–0.07	178 (0.08)	0.07–0.09
*P*	< 0.001		< 0.001		< 0.001		< 0.001	

*HPV* Human papillomavirus, *HR-HPV* High-risk HPV, *LR-HPV* Low-risk HPV, *CI* Confidence interval, *NA* Not applicable

## Discussion

Here we measured the prevalence of 21 distinct HPV types and found the overall prevalence of HPV in this representative sample of women was 18.71% and HR-HPV of 15.17%, amongst 214,715 women prior to HPV vaccination. The high HR-HPV prevalence indicated the inadequacy of routine cervical screening in the region. Additionally, the large sample size allows for precise estimation of both increases and decreases in HPV type specific prevalence, which could be used as a baseline of comparison to future sampling of the entire population.

Global HPV prevalence estimates are known to vary by the region, study design, target population and calendar time [16]. According to the previous reports, HPV positive rates range from 6.70 to 44.50% in China [11]. Notably, Western Asia (1.70%) and North America (4.70%) had a low rates of HPV infection, while East Africa (33.6%) and the Caribbean (35.4%) had a high rates of HPV infection [17–19]. HR-HPV genotype distribution was also heterogeneous, ranging from 9.90–27.50% in China, which is 15.17% in current study, as with that in Guangdong (20.02%), Guiyang (20.45%) and Nanning (22.28%) [20]. The variation was expected to be explained by cultural diversity, the sampling strategy, methods and devices, as well as the sensitivity and specificity of the HPV detection assays.

In present study, the sampling methods and devices, and Hybribio test were enrolled for HPV detection assays unaltered for over 7 years, which could monitor the annual changes of HPV prevalence sufficiently. We found HPV prevalence showed a significant downward trend from 2012 to 2016. Analogous results of the HR-HPV positive rates were also found to decline from 25.3% in 2007 to 18.4% in 2014, in Guangzhou [21]. This decline may be due to the following reasons: 1) emphasis on cervical cancer has led to increase participation in screening, including those without cervical abnormalities; 2) in the last decade, some women tested positive for HPV became negative, due to immunization and treatment; and 3) individuals were more likely to be aware of HPV and HPV vaccination following the continuous improvements of living conditions and public health awareness. Several studies have demonstrated the association of HPV with economic development. Women from impoverished countries and areas suffered a high prevalence of HPV, e.g., 66.7% among young females in South Africa and 44.5% in Henan province of China [19, 22]. On the contrary, a low rate of 6.7% was found among women in Beijing—the capital of China, which remained the most flourishing cultural and economic center in China, and also had the excellent healthcare system, indicating a strong correlation of HPV with socioeconomic development [23].

We found HPV52 was the most commonly detected genotype, in agreement with that stated in Japan, Taiwan, and eastern Africa [24]. However, it was inconsistent with the data reported that HPV16 was the predominant genotype in other studies [3] and HPV35 in sub-Saharan Africa [25]. Moreover, prevalence of both HPV52 (5.12%) and − 58 (2.51%) was higher than that reported (2.3 and 1.0% respectively) in the United States [3], and a nationwide population-based investigation in 37 cities in China [20]. HPV 16 is the strain most likely to cause cancer. Thus, clinic-based studies usually found higher prevalence of HPV16 than population-based studies [24, 26]. We found a relatively low prevalence of HPV16, which was in accordance with an estimated HPV16 prevalence of 3.2% from a meta-analysis of 1 million women with normal cytology [27]. It is possible that most women seemingly healthy from the physical examination center enrolled in our study were more likely to detect HPV types not related to cervical infection. Our data also indicated that HPV52, − 16, and − 58 were consistently the top three HR-HPV genotypes from 2012 to 2018, suggesting the HPV vaccine covering these HR-HPV types is routinely recommended, especially for those females at young ages exposed to HPV in this region.

Evaluations in the United State showed that young women had the highest HPV prevalence [3], consistent with our the findings of the highest HPV prevalence (33.11%) among women aged less than 21 years. Young women often have a high infection rate, mainly because they are sexually more active before their immune systems become less sensitive [17]. Although they were known to have high risks for HPV infection, however it is temporary and supposed to disappear within a year or two, and thus its prevalence declined gradually with respect to ages [18]. Predictably, the prevalence of HPV slightly declined in middle ages, yet significantly increased among the oldest people, which are consistent with those in most developed countries and the data from Bruni and colleagues [27]. The mechanism of this increase in infection rates is unclear at present. Other than persistent infections that seem to be more prominent among females at older ages [19], this increase could be also explained by re-marriage, reactivation of latent HPV in menopausal women and the cohort variation [16]. In addition, women aged 31–40 years were the most common population for screening, and those aged 41–50 years showed an increase trend in this study, thus routine screening is strongly recommended for women over 30.

In an unweighted analysis of women with multiple HPV genotype infections, we found HR-HPV genotypes accounted for over 80% of multiple infections, which mainly occurred at ages either younger than 21 or older than 60. Some have suggested HPV infection with multiple genotypes may prolong the duration of infection and increase the risk of cervical cancer and cervical precancerous lesions [28], leading to a complication of multiple infections among older women. Multiple infections were believed to have competitive and/or cooperative interactions between HPV genotypes [29]. Although the mechanisms and potential oncogenic effects of multiple genotype infections still require further investigation, this study could still be beneficial to the development of HPV prophylactic vaccines.

The overall strengths of this study include the large sample size, unaltered HPV genotyping methods over 7 years, decade-long study period measuring the trends of HPV infection on a yearly basis, and the use of PCR testing rather than serologic tests, which allows for accurate determination of simultaneous co-infection, all of that provide a good baseline for epidemiological surveillance after the introduction of HPV vaccine at population level. However, several limitations exist in our study. Firstly, HPV DNA testing does not reflect the previous and cumulative incidence of HPV infections, but only the current infection status. Secondly, our study included specimens from women, without pathological data, such as, cervical cytology and histology results, which was unable to explain the relationship between HPV infection and pathology. Thirdly, no male samples were analyzed, so our study would not be able to represent the infection of HPV in general population in the region. Lastly, the detailed information about the patients, such as education level, economic status and background related to HPV infection, were not documented in this study, hindering a more comprehensive evaluation of the effects of these factors on the prevalence of HPV infection.

## Conclusions

In conclusion, our study provides an estimate of HPV infection among a large female population in Southern China. Overall, HPV prevalence was high (18.71%), showing a downward trend from 2012 to 2018, and women aged less than 21 years old showed the highest HPV prevalence. Importantly, HPV52, − 16, and − 58 were the major genotypes at all times, regardless of the fact that their ranks varied with respect to the ages and years. Our study enables the estimations of HPV vaccine impact among women spanning a broad age range, provide guidance for clinical care and public health policy including cervical screening and vaccination, and would also be useful for the other low- and middle-income areas with a heavy HPV infection burden to fight against cervical cancer.

## Data Availability

The datasets used and/or analysed during the current study are available from the corresponding author on reasonable request.

## Abbreviations

HPV: Human papillomavirus
HR-HPV: High-risk HPV
LR-HPV: Low- risk HPV
CI: Confidence interval
